# Diagnosis and treatment of a diaphragmatic pheochromocytoma: A case report

**DOI:** 10.1016/j.ijscr.2020.04.018

**Published:** 2020-05-07

**Authors:** Xiangan Wu, Bao Jin, Shi Chen, Shunda Du, Yilei Mao, Xinting Sang

**Affiliations:** a Department of Liver Surgery, Peking Union Medical College Hospital, Chinese Academy of Medical Sciences and PUMC, Beijing, China; b Department of Endocrinology, Peking Union Medical College Hospital, Chinese Academy of Medical Sciences and PUMC, Beijing, China

**Keywords:** Diaphragm paraganglioma, MIBG, Preoperative preparation, Surgery

## Abstract

**Background:**

Ectopic pheochromocytomas, the incidence of which is >15%, can occur throughout the entire body but seldom on the diaphragm. Surgery may the first-choice treatment for ectopic pheochromocytomas.

**Presentation of case:**

We herein describe a 61-year-old woman with an atopic diaphragmatic pheochromocytoma. She had a 7-year history of paroxysmal headaches, palpitations, and hypertension with no obvious causes; these symptoms were alleviated by nifedipine and metoprolol. Computed tomography (CT) revealed a slightly hypodense lesion on top of the right hepatic lobe. A Metaiodobenzylguanidine (MIBG) scan showed increased radioactive in the lesion. After adequate preoperative preparation, we removed the mass. During the operation, we found that the mass was located on the diaphragm. The pathological examination showed that the main pathologic change was paraganglioma. The patient recovered well after surgery with no recurrence of her hypertension, palpitation, or headache.

**Conclusions:**

Diaphragmatic pheochromocytoma is a rare kind of ectopic pheochromocytomas, which can also affect the patient’s quality of life. Combination of qualitative and positioning tests can assist in diagnosis of ectopic pheochromocytoma. Surgical resection is an effective treatment method.

## Highlights

• Ectopic pheochromocytoma seldom occurs on the diaphragm and is hard to diagnose.
• Combination of catecholamine, CT and especially MIBG can assist in diagnosis of ectopic pheochromocytoma.
• Surgery may the first-choice treatment for ectopic pheochromocytomas.

## 1 Introduction

Ectopic pheochromocytomas that arise from chromaffin cells of the sympathetic ganglia are referred to as catecholamine-secreting paragangliomas or extra-adrenal pheochromocytomas. These tumors can occur throughout the entire body, especially the para-aortic region, renal hilus, and inferior vena cava. They are relatively rare on the base of skull, heart, gastrointestinal system, sacroiliac blood vessels, ureter, uterus, and ovaries [[1], [2], [3]]. To our knowledge, only two cases of diaphragmatic pheochromocytomas with a simple clinical course have been reported [4,5]. We herein describe the typical clinical course of a pheochromocytoma on the right diaphragm that was misdiagnosed as a liver tumor. This work has been reported in line with the SCARE criteria [6].

## 2 Presentation of case

A 61-year-old woman presented to our institute for evaluation of a liver mass. She had a 7-year-history of paroxysmal headache and palpitation. During a routine physical examination, an approximately 3-cm liver mass had been found by ultrasonography, and a hepatic hemangioma was suspected. She reported intermittent symptoms of headache, palpitation, and hypertension up to 200/110 mmHg with no obvious causes since 2007. These symptoms were alleviated after taking nifedipine and metoprolol. The symptoms thereafter resolved, and she stopped the treatment herself. In September 2013, the symptoms recurred and were frequently aggravated with postural hypotension. The patient had a 7-year history of diabetes mellitus, a 3-year history of cholecystolithiasis, and a 6-month history of hyperlipidemia. She presented to our hospital for further diagnosis and treatment. Physical examination revealed a body temperature of 36.5 °C, pulse of 70 bpm, standing blood pressure of 120/80 mmHg, and supine blood pressure of 140/100 mmHg. Cardiopulmonary and abdominal examinations showed no obvious abnormalities.

**Fig. 1:**
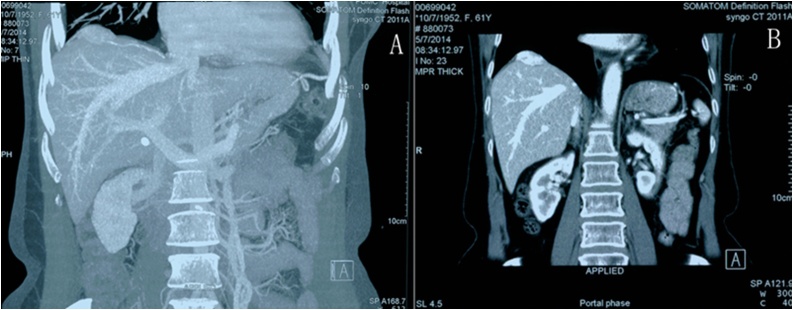
Computed tomography shows an arched lesion on the top of the liver.

Considering that these symptoms are consistent with pheochromocytoma, we ran many tests. The patient’s blood norepinephrine level was high, and her 24-h urine epinephrine, norepinephrine, and dopamine levels were 2.66, 63.94, and 130.54 μg, respectively. Thoracic and abdominal enhanced CT (Fig. 1) revealed a 27.8 × 43.4 mm slightly hypodense lesion on top of the right hepatic lobe with vivid inhomogeneous enhancement. An MIBG scan showed increased radioactivity uptake of the lesion, and an ectopic pheochromocytoma could not be excluded. Somatostatin receptor imaging showed no abnormalities, and an electrocardiogram showed sinus rhythm. However, when testing the erect and supine aldosterone level, the patient developed palpitation and dizziness. At this time, an electrocardiogram showed atrial tachycardia of 109 bpm, probably with atrial fibrillation and differential pacing. However, all of these manifestations disappeared within 40 min. The electrocardiogram became normal. An ultrasonic cardiogram and coronary CT angiogram also showed no abnormalities.

Our presumptive diagnosis was liver paraganglioma based on the clinical manifestations and test results. We initiated treatment with phentolamine at 5 mg twice a day; this was gradually increased to 27.5 mg/day. We monitored the patient’s erect and supine blood pressure, heart rate, weight, and intake–output fluid volume. After initiating treatment, her blood pressure stabilized and she gained 6 kg of body weight. We then performed an operation on 10 June 2014. During surgery, we found that the tumor was protruding from the diaphragm. The tumor had a diameter of 4 cm, and soft pitting was present on top of the liver. A small amount of yellow moss was stuck to the external surface of the soft/solid tumor, which was vascularized. The patient’s blood pressure increased when the tumor was touched. We sliced the diaphragm with a 0.5-cm margin around the tumor by electrocoagulation and sewed the wound closed. Before suturing the diaphragm, we filled her lung to exhaust the air in the chest and checked in water. We placed a drainage tube under the diaphragmatic wound and returned the patient to the intensive care unit. The patient recovered well; her symptoms of paroxysmal hypertension and palpitations disappeared, and the catecholamine levels in her blood and urine returned to normal.

**Fig. 2:**
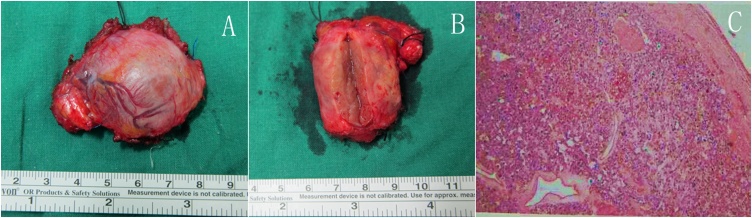
Specimen and pathology. (A,B) show the whole mass and cross section of the mass. (C) shows the pathologic image of the mass and pathologic findings are as follows: CgA(+), Melan-A(−), S-100(+), Syn(+), Vimentin(+), p53(−), AE1/AE3(−), Calretinin(−), ɑ-inhibin(−), Ki-67(index<1).

The final pathological examination (Fig. 2) suggested that the main pathologic change was paraganglioma (ectopic pheochromocytoma).

Several weeks before the present writing, she underwent outpatient follow-up and was still clinically well without recurrence.

## 3 Discussion

The incidence of ectopic pheochromocytoma is >15%, and both the location and clinical presentation are variable [7]. Consequently, the clinical diagnosis and treatment are difficult, especially for tumors that develop as a small hidden focus.

The typical clinical manifestations of pheochromocytoma are paroxysmal hypertension, headache, palpitations, and excessive perspiration [8]. In the present case, the patient showed typical clinical symptoms of pheochromocytoma. However, her condition was not diagnosed until 7 years later, at which time the ectopic site was detected. When a patient presents with typical symptoms of pheochromocytoma, the possibility of both pheochromocytoma and paraganglioma should be considered. We can then examine the patient’s blood and urine catecholamine levels or metabolite contents, such as metanephrine or normetanephrine [9]. The qualitative diagnosis may not be difficult when elevated urine vanillylmandelic acid and catecholamines are present.

The most difficult aspect of the diagnosis of pheochromocytoma or paraganglioma is the positioning diagnosis. CT, magnetic resonance imaging, and MIBG scans have great value in the locative diagnosis [8,10]. In particular, the MIBG scan has high sensitivity and specificity and thus plays a very important role in both the qualitative and locative diagnosis [11,12]. In the present case, the elevated norepinephrine level suggested a possible pheochromocytoma, and CT revealed a suspicious lesion on top of the right lobe of the liver with no obvious abnormalities of the bilateral adrenal glands. The MIBG scan confirmed an area of increased radioactive uptake at the top of the right hepatic lobe, and we preliminarily diagnosed the patient with hepatic paraganglioma. In addition, for patients with symptoms suggestive of heart disease, tests such as electrocardiography, ultrasonic cardiography, and coronary CT angiography should be performed for diagnosis. Our patient showed no abnormalities of the function or structure of her heart.

Surgery may be the only way to cure this disease [13]. Preoperative preparation involving plasma volume expansion, blood pressure monitoring, heart rate monitoring, and anesthesia planning is necessary. Such preparation could reduce the perioperative mortality [10,14]. To ensure that the operation is smoothly performed, we used the α-adrenergic receptor blocker phentolamine to control the blood pressure, reduce the heart load, and supply an adequate blood volume [15]. Pheochromocytoma is associated with a risk of a sudden increase in the intraoperative blood pressure as well as perioperative hypotension and shock. Before surgery in the present case, the anesthesiologist performed the central venipuncture, monitored the arterial blood pressure, and prepared the vasoactive agents. We used a costal margin incision in the right upper quadrant to allow for full visualization of the tumor. We then located the tumor in the right diaphragm. An incision with a 0.5-cm margin was carefully made around the tumor to avoid stimulating it. We closely communicated with the anesthesiologists during this procedure. According to the intraoperative monitoring situation, the anesthesiologists can adjust the blood pressure and rapidly expand the plasma volume with crystalloids and colloids in a timely manner [16,17]. During the operation, the patient’s blood pressure suddenly increased to 201/165 mmHg when we stimulated the muscle surrounding the tumor. However, the pressure stabilized soon after administration of a decompression drug and remained stable until the end of the operation.

The main risk in the postoperative period is hypotension [7,10]. Thus, the blood pressure should be monitored, the infusion speed should be adjusted in a timely manner, and booster drugs should be given when necessary. Our patient was sent to the intensive care unit after the operation, and her vital signs became stable. In our opinion, patients benefit from taking phentolamine and metoprolol to control their blood pressure and heart rate before surgery.

## 4 Conclusion

In conclusion, Diaphragmatic pheochromocytoma is a rare kind of ectopic pheochromocytomas, which can cause various symptoms and affect the patient’s quality of life. A qualitative diagnosis can be made by the presence of typical symptoms combined with blood and urine examination of catecholamines. The positioning diagnosis can be performed by medical imaging examinations such as CT and especially MIBG scan. Surgical resection is an effective treatment method, but it requires adequate preparation before surgery, strict intraoperative monitoring, and careful management after surgery.

## Conflicts of interest

None.

## Sources of funding

None.

## Ethical approval

The study is exempt from ethnical approval in our institution.

## Consent

Written informed consent was obtained from the patient for publication of this case report.

## Author contribution

Xiangan Wu: Data curation, Writing - Original draft.

Bao Jin: Investigation.

Shi Chen: Resources.

Shunda Du: Writing - Review & Editing, Project administration.

Yilei Mao, Xinting Sang: Supervision.

## Registration of research studies

Not acquired.

## Guarantor

The correspondent author.

## Provenance and peer review

Not commissioned, externally peer-reviewed.
